# Hydrophobicity and Aromaticity Are Primary Factors Shaping Variation in Amino Acid Usage of Chicken Proteome

**DOI:** 10.1371/journal.pone.0110381

**Published:** 2014-10-16

**Authors:** Yousheng Rao, Zhangfeng Wang, Xuewen Chai, Qinghua Nie, Xiquan Zhang

**Affiliations:** 1 Department of Biological Technology, Nanchang Normal University, Nanchang, Jiangxi, China; 2 Guangdong Provincial Key Laboratory of Agro-animal Genomics and Molecular Breeding, South China Agricultural University, Guangzhou, Guangdong, China; Wageningen UR Livestock Research, Netherlands

## Abstract

Amino acids are utilized with different frequencies both among species and among genes within the same genome. Up to date, no study on the amino acid usage pattern of chicken has been performed. In the present study, we carried out a systematic examination of the amino acid usage in the chicken proteome. Our data indicated that the relative amino acid usage is positively correlated with the tRNA gene copy number. GC contents, including GC1, GC2, GC3, GC content of CDS and GC content of the introns, were correlated with the most of the amino acid usage, especially for GC rich and GC poor amino acids, however, multiple linear regression analyses indicated that only approximately 10–40% variation of amino acid usage can be explained by GC content for GC rich and GC poor amino acids. For other intermediate GC content amino acids, only approximately 10% variation can be explained. Correspondence analyses demonstrated that the main factors responsible for the variation of amino acid usage in chicken are hydrophobicity, aromaticity and genomic GC content. Gene expression level also influenced the amino acid usage significantly. We argued that the amino acid usage of chicken proteome likely reflects a balance or near balance between the action of selection, mutation, and genetic drift.

## Introduction

Codons and amino acids are utilized with different frequencies both among species and among genes within the same genome. Such biases in codon and amino acid usage have been studied extensively in a variety of organisms. Although the genetic code is degenerate, meaning that it can use different combinations of codons to make the same protein, the processes that shape nonrandom usage of codons also have the potential to influence the amino acid usage in proteins. This can be attributed to neutral processes as the base compositions of all the codons encoding a given amino acid may be GC rich or GC poor [1]. In addition, selection may play an important role in shaping amino acid frequencies because functional similar amino acids may have different tRNA abundances or require different metabolic costs for their production [2]–[8]. The genomic base composition generally has significant impact on the amino acid usage pattern [1], [9]–[15]; likewise, amino acid usage has also been shown to be influenced by other factors such as hydrophobicity, aromaticity, cysteine residue (Cys) content, gene function, mean molecular weight and expression level etc. [10], [13], [16]–[17].

Chicken (*Gallus gallus*) is an important model organism that bridges the evolutionary gap between mammals and non-amniote vertebrates, and is by far the best studied representative of all avian species. The assembly of the chicken genome, with its distinctive sequence features, reinforced isochore structure and organization provides an ideal model to explore some important issues in genome structure and evolution [18]–[20]. Recently, Rao et al. [21] carried out a systematic examination of the codon usage in chicken, suggesting that both mutation and selection are involved in shaping the codon usage, however, the driving force is the mutation bias. Up to date, no study on the amino acid usage pattern of chicken has been performed. The aim of this study is to explore this issue, and to describe general trends and their biological implications.

## Materials and Methods

### Sequence data

In this study, only nuclear genes with complete information on protein-coding sequence and with no evidence of multiple-splicing forms were included. Gene sequences were downloaded from the NCBI RefSeq database (ftp://ftp.ncbi.nlm.nih.gov/genomes/Gallus_gallus/). Coding DNA sequences (CDSs) and complete mRNA sequences (or full length cDNA sequences) corresponding to all annotated genes in the chicken genome was downloaded from Ensembl. For genes with a single splicing isoform, CDS length should be equal to the total length of all exons. Thus, we discarded all sequences showing a length difference of at least one base. Genes with a CDS that did not begin with an ATG start codon, or did not have a length ≥ 300 bp, or did not occur in multiples of three nucleotides, or contained an internal stop codon, were also discarded.

### Expression data and tRNA gene copy number data

Chicken expression data was taken from a previous work [20], in which data for 19 tissues of blood, brain, bursa of fabricius, cecum, connective tissue, embryonic tissue; epiphyseal growth plate, gonad, head, heart, limb, liver, muscle, ovary, pancreas, spleen, testis, and thymus are included. Two indices, expression level and expression breadth, were used to measure the expression pattern of genes. For a given gene, expression level is the number of EST counts in all tissues, and expression breadth is the number of tissues in which ESTs are found.

The tRNA gene copy numbers for each codon in the *G. gallus* genome were taken from http://gtrnadb.ucsc.edu/Ggall/. In these data, pseudogenes have already been removed.

### Correspondence analysis

Correspondence analysis (COA) as implemented by CodonW 1.4.2 was used to determine the major factors shaping variation in amino acid usage among chicken proteins. For each gene, the relative amino acid usage (RAAU), the GC content of the CDS (GC_cds_), the GC content at the first, second and third position (GC1, GC2 and GC3), the average hydrophobicity and aromaticity, were calculated by codonW 1.4.2. The PHD software (http://npsa-pbil.ibcp.fr/cgi bin/npsa_automat.pl?page = /NPSA/npsa_phd.html) was used to predict the protein secondary structure, including alpha helix, extended strand, and random coil.

### Statistical analysis

Correlation analysis between variables was performed by SAS Proprietary Software Release 8.1. In order to assess the actual strength of association, all correlation coefficients reported in this study was obtained using all genes independently. The significance tests were corrected for multiple testing by the Bonferroni step-down correction [22].

## Result

### Relationship between the relative amino acid usage and the tRNA gene copy number

**Figure 1:**
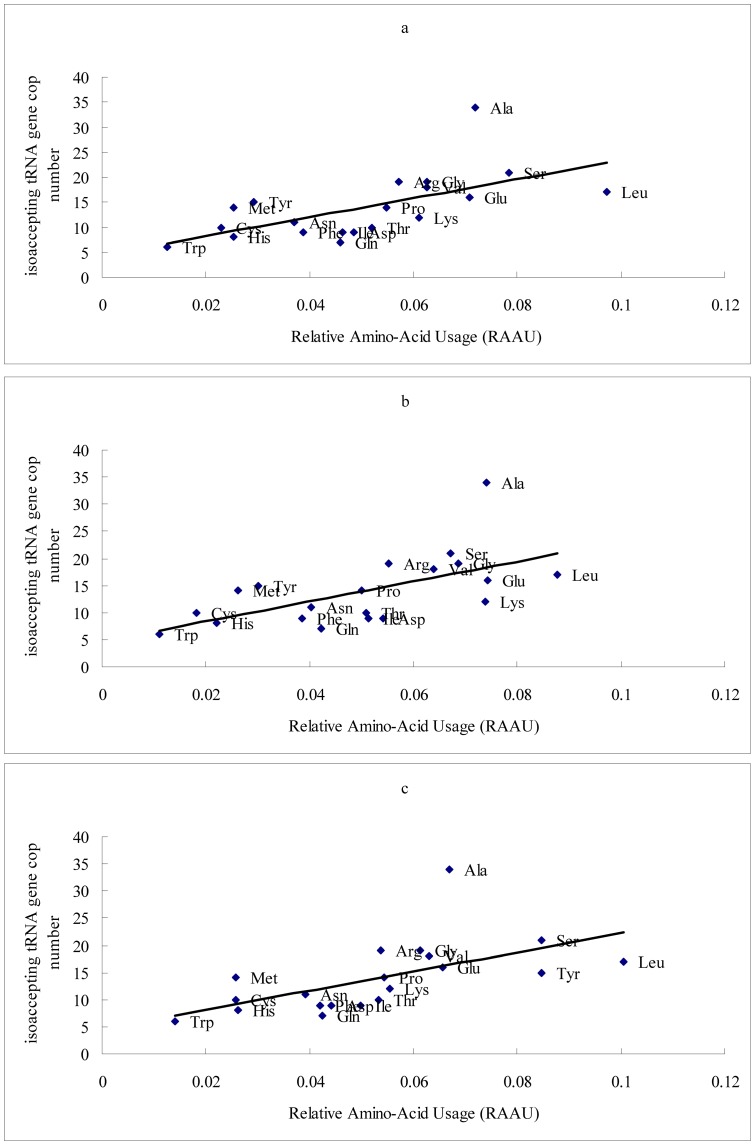
Relationship between the relative amino acid usage and the tRNA gene copy number. The sequence collection contained 8631 CDSs, each corresponding to a unique gene in the *Gallus gallus* genome. We used 5% of the total genes with extremely high and low expression levels inferred from EST counts, as the high and low expression data set, then calculated the relative amino acid usage (RAAU) for the total data set, high expression data set and low expression data set, respectively. The tRNA gene copy numbers for each codon in the *G. gallus* genome was taken from http://gtrnadb.ucsc.edu/Ggall/. The isoaccepting tRNA genes were summed for each amino acid. The average RAAU values of three samples correlated with the isoaccepting tRNA gene copy number significantly. a. Relationship between the average RAAU of the total genes with the tRNA gene copy number (r  =  0.6215, P <0.0001); b. Relationship between the average RAAU of the highly expressed genes with the tRNA gene copy number (r  =  0.6578, P <0.0001); c. Relationship between the average RAAU of the lowly expressed gene with the tRNA gene copy number (r  =  0.5928, P <0.0001).

In this study, only nuclear genes with complete information on protein-coding sequence and with no evidence of multiple splicing forms were included. The sequence collection contained 8631 CDSs, each corresponding to a unique gene in the *G. gallus* genome. We used 5% of the total genes with extremely high and low expression levels inferred from EST counts, as the high and low expression data set, and then calculated the relative amino acid usage (RAAU) for the total data set, high expression data set and low expression data set, respectively. The tRNA gene copy numbers for each codon in the *G. gallus* genome was taken from http://gtrnadb.ucsc.edu/Ggall/. The isoaccepting tRNA genes were summed for each amino acid. ΔRAAU for a given amino acid was defined as the difference between the average RAAU of genes with high and low expression level (significance tested using the one-way analysis of variance (ANOVA) by SAS). Relationships between the relative amino acid usage and tRNA gene copy numbers for chicken genes with the total, the highly expressed and the lowly expressed were shown in figure 1. The average RAAU values of three samples are correlated with the isoaccepting tRNA gene copy number significantly (RAAU of the total vs. tRNA gene copy number, r  =  0.6215, P <0.0001; RAAU of the highly expressed vs. tRNA gene copy number, r  =  0.6578, P <0.0001; RAAU of the lowly expressed vs. tRNA gene copy number, r  =  0.5928, P <0.0001).

Table 1 showed that the amino acids are not equally used in the chicken genome. The content of Leu, Glu, Gly, Lys, Ser, Val and Ala in the chicken proteins are significantly high, otherwise, some amino acid content such as Cys, His, Met, Tyr are significantly low. Amino acids that are significantly overrepresented in the highly expressed chicken genes are Ala, Asp, Glu, Gly and Lys, which are not aromatic and less energetic cost. Amino acids that are significantly overrepresented in the lowly expressed chicken genes are Cys, His, Leu, Phe, Pro, Ser, Thr, Trp and Tyr. Three aromatic amino acids are included in this group. According to Palacios et al [23], we divided amino acids into "GC rich" and "AT rich" categories based on the GC content of codons. Although a most recent study demonstrated that both GC_cds_ and GC3 are weakly correlated with the gene expression level in chicken [24], table 1 did not show any specific distribution for these two types amino acids among the highly expressed data set and the lowly expressed data set.

**Table 1:** Amino acid usage in chicken proteins.

AA	Codon	GC- rich	AT- rich	Aromatic	RAAU Total	RAAU High	RAAU Low	ΔRAAU
Ala	GCN	+			0.0720	0.0741	0.0668	0.0073***
Arg	CGN; AG(AG)	+			0.0572	0.0552	0.0538	0.0014
Asp	GA(TC)				0.0485	0.0541	0.0443	0.0098***
Asn	AA(TC)		+		0.0371	0.0403	0.0392	0.0011
Cys	TG(TC)				0.0229	0.0181	0.0258	−0.0077***
Gln	CA(AG)				0.0459	0.0423	0.0424	−0.0001
Glu	GA(AG)				0.0708	0.07430	0.0655	0.0088***
Gly	GGN	+			0.0625	0.0687	0.0612	0.0075***
His	CA(TC)				0.0253	0.0220	0.0262	−0.0042***
Ile	AT(TCA)		+		0.0464	0.0513	0.0500	0.0013
Leu	CTN, TT(AG)		+		0.0972	0.0878	0.1003	−0.0125***
Lys	AA(AG)		+		0.0610	0.0740	0.0555	0.0185***
Met	ATG				0.0253	0.0263	0.0258	0.0005
Phe	TTT, TTC		+	+	0.0387	0.0386	0.0419	−0.0033**
Pro	CCN	+			0.0548	0.0500	0.0543	−0.0043*
Ser	TCN; AG(TC)				0.0785	0.0671	0.0848	−0.0177***
Thr	ACN				0.0520	0.0509	0.0532	−0.0023
Trp	TGG	+		+	0.0126	0.0111	0.0141	−0.0030***
Tyr	TA(TC)		+	+	0.0292	0.0301	0.0848	−0.0547***
Val	GTN				0.0627	0.0639	0.0630	0.0009

The relative amino acid usage (RAAU) for the total data set, high expression data set and low expression data set were calculated, respectively. ΔRAAU for a given amino acid was defined as the difference between the average RAAU of genes with high and low expression [significance tested using the one-way analysis of variance (ANOVA) by SAS]. * represents P <0.05; ** represents P <0.01; *** represents P <0.0001.

### Impact of GC content on amino acid usage

Many studies have documented that the usage of amino acid types encoded by codons rich or poor in GC content is correlated with the genomic GC content significantly [1], [ 9]–[15]. This trend also existed in chicken. As shown in table 2, the RAAU is positively correlated with GC1, GC2, GC3 and GC_cds_ for GC rich amino acids such as Pro, Ala, Arg and Gly, otherwise, the RAAU is negatively correlated with GC1, GC2, GC3 and GC_cds_ for GC poor amino acids such as Phe, Ile, Asn and Lys. For other intermediate GC content amino acids, the RAAU shows either positive/negative or no significant trend with them. We also retrieved all intronic sequences for each gene and provided the combined length of all introns for a gene exceeding 200 bp and calculated the GC content of the intronic sequences (GC _introns_). Regression analyses demonstrated that GC _introns_ correlated with the RAAU for Ala, Arg, Asp, Asn, Glu, Gly, Ile, Lys, Pro, Thr, Trp and Tyr, but with much lower coefficients than GC1, GC2, GC3 and GC_cds_. In order to determine this 5 variables contributing to amino acid usage and how they may interact, we performed multiple linear regressions with these variables, excluding those not contributing significantly through the use of the t-statistical logarithm with backward stepwise regression. Regression analyses indicated that the adjusted R-square for Pro, Ala, Arg and Gly is 0.4001, 0.3098, 0.1166 and 0.2884, respectively. The best combinations of variables were GC1 and GC2 for Ala, Arg, Gly, and GC2, GC_cds_ for Pro. The adjusted R-square for Phe, Ile, Asn and Lys is 0.1711, 0.2885, 0.2380 and 0.2237, respectively. The best combinations of variables were GC1 and GC2 for Phe, Ile, Asn, and GC2, GC_cds_ for Lys. For other intermediate GC content amino acids, the R-square is range from 0.0067 to 0.3331 (see table 2). The vertebrate genome comprises a mosaic of long stretches of GC rich and AT rich regions, the so-called isochore structure [25]. Although a study suggested that the GC content is becoming homogenized in humans [26], Webster et al. [19] found that the heterogeneity in the GC content is being reinforced in the chicken genome. In order to test whether this heterogeneous distribution of GC content has any significant impact on the amino acid usage in chicken, we produced a high GC3 sample (20% of the highest GC3 of the CDSs) and a low GC3 sample (20% of the lowest GC3 of the CDSs) and compared the relative amino acid usage between two datasets using the one-way analysis of variance (ANOVA) by SAS. Here, we used GC3 variation as a surrogate for isochore structure because GC3 is strongly correlated with intronic GC content in this data set (r  =  0.7713, P <0.0001) [25]. Our data indicated that, except for Tyr, Gln, Phe, the RAAU for other amino acids in GC rich isochore significantly differs from that in GC poor isochore. Leu, Met, Val, Pro, Ala, His, Cys, Trp, Arg and Gly are over presented in the GC rich isochore, otherwise, the Ile, Ser, Thr, Gln, Asn, Lys, Asp and Glu are over presented in the GC poor isochore. To further test this result, we also used the GC content of the surrounding regions of gene (25 kb upstream of the initiation codon plus the 25 kb downstream of the stop codon) as an estimator for isochore structure according to Sabbía et al. [13], and found that, except for Gln and Leu, the RAAU for other amino acids in GC rich isochore significantly differ from that in GC poor isochore. Although there are slight differences between two estimators, our data clearly demonstrated that the isochore structure has significant impact on the amino acid usage in the chicken genome.

**Table 2:** Relationship between GC content and the relative amino acid usage in chicken.

AA	Codon	GC- rich	AT- rich	GC1 vs. RUUA	GC2 vs. RUUA	GC3 vs. RUUA	GC_cds_vs. RUUA	GC_intron_vs. RUUA	Adjusted R^2^
Ala	GCN	+		0.4762	0.3938	0.2317	0.4189	0.1707	0.3098
Arg	CGN;AG(AG)	+		0.2109	0.3021	0.2080	0.2991	0.1393	0.1166
Asp	GA(TC)			0.0772	−0.3408	−0.1675	−0.2018	−0.0704	0.1692
Asn	AA(TC)		+	−0.4273	−0.3303	−0.2902	−0.4263	−0.2106	0.2380
Cys	TG(TC)			−0.1974	0.2660	0.1021	0.0989	0.0346*	0.1705
Gln	CA(AG)			0.2168	−0.1440	−0.0325*	−0.0067*	−0.0266*	0.1007
Glu	GA(AG)			0.2070	−0.4183	−0.1384	−0.1692	−0.0653	0.3063
Gly	GGN	+		0.3429	0.4898	0.1477	0.3526	0.1195	0.2884
His	CA(TC)			0.0693*	−0.0144*	0.0504*	0.0503*	0.0208*	0.0067
Ile	AT(TCA)		+	−0.4410	−0.4244	−0.2152	−0.4070	−0.1705	0.2885
Leu	CTN, TT(AG)		+	0.0957	−0.3084	0.1270	0.0196*	0.0315*	0.1514
Lys	AA(AG)		+	−0.2441	−0.4197	−0.2589	−0.3808	−0.1477	0.2237
Met	ATG			−0.1733	−0.1919	0.0526	−0.0718	0.0250*	0.0844
Phe	TTT, TTC		+	−0.3275	−0.2619	−0.0025*	−0.1755	−0.0257*	0.1711
Pro	CCN	+		0.3306	0.5895	0.1187	0.3599	0.1105	0.4001
Ser	TCN; AG(TC)			−0.2481	0.4460	−0.0577	0.0289	−0.0360*	0.3331
Thr	ACN			−0.3309	0.2092	−0.0996	−0.0975	−0.0525	0.1955
Trp	TGG	+		−0.1741	0.1652	0.1217	0.0877	0.0674	0.1239
Tyr	TA(TC)		+	−0.2866	−0.2038	0.0013*	−0.1432	−0.1033	0.1237
Val	GTN			0.0790	−0.1626	0.0601	0.0136*	0.0404*	0.0462

For each gene, the relative amino acid usage (RAAU), the GC content of the CDS (GCcds), the GC content at the first, second and third position (GC1, GC2 and GC3), were calculated by codonW 1.4.2. We also retrieved all intronic sequences for each gene and provided the combined length of all introns for a gene exceeding 200 bp and calculated the GC content of the intronic sequences (GC _introns_). The linear regression analyses were made between RAAU and GCcds, GC1, GC2, GC3 and GC _introns_ for each amino acid. The regression coefficients were presented on the table. In order to determine this 5 variables contributing to the amino acid usage and how they may interact, we performed multiple linear regressions with these variables, excluding those not contributing significantly through the use of the t-statistical logarithm with backward stepwise regression. The adjusted R-square for each amino acid was given in the last column. * represents P>0.05.

### Correspondence analysis for amino acid usage

**Figure 2:**
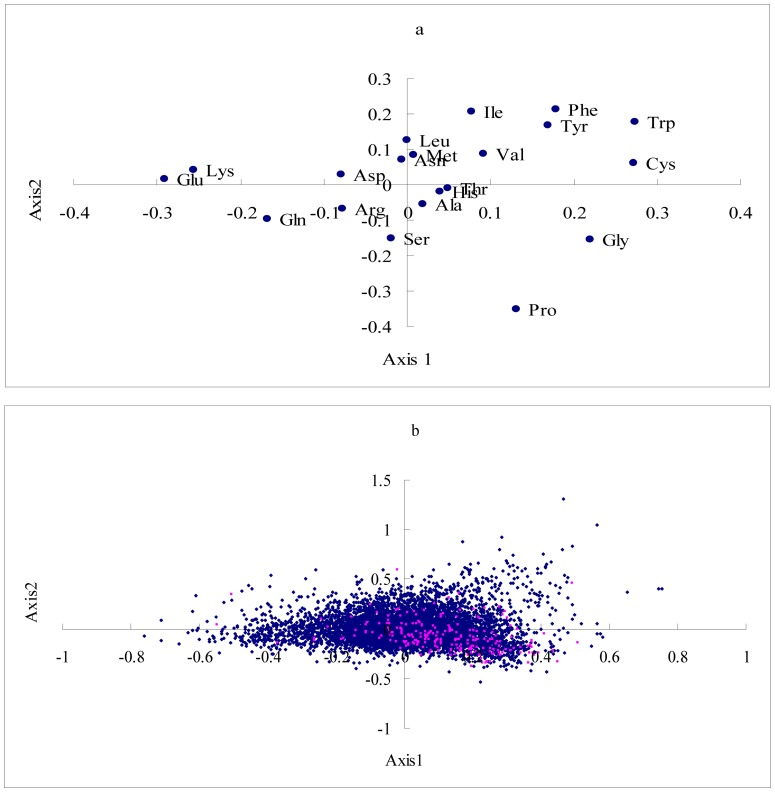
Distribution of the amino acids and genes on the first two axes of the correspondence analyses. a. Representation of the first two axes of the correspondence analysis performed on the amino acid frequencies of the chicken protein. b. Representation of the first two axes of the correspondence analysis performed on the amino acid frequencies of 8631 chicken genes. Membrane proteins are indicated by red dots.

**Figure 3:**
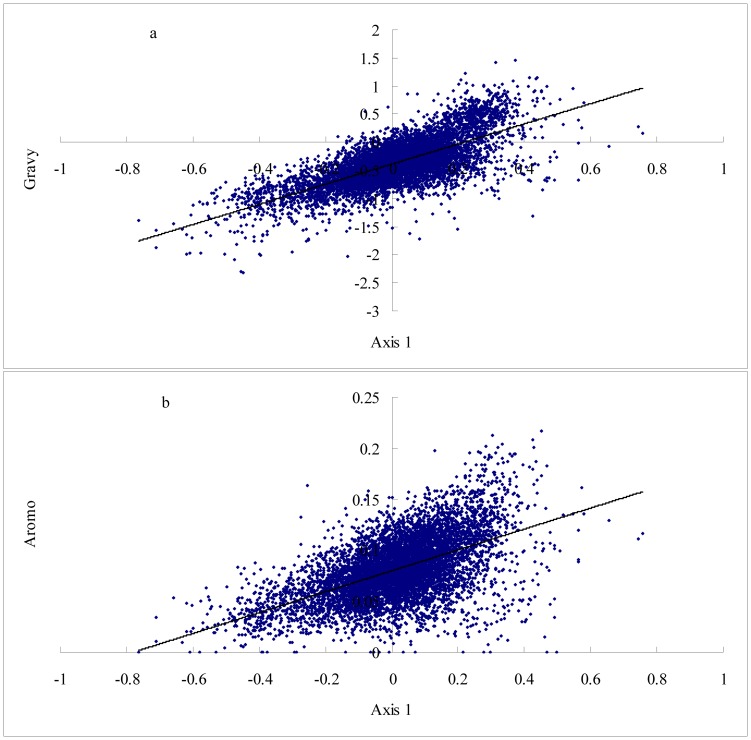
Relationship between Axis 1 and the GRAVY score of proteins, the Aromo score of proteins. a. Axis 1 is strongly correlated with the GRAVY score of proteins (r  =  0.7341, P <0.0001); b. Axis 1 is strongly correlated with the Aromo score of proteins (r  =  0.5519, P <0.0001).

A correspondence analysis of the amino acid usage of this data indicated that 4 of the 19 axes account for almost 50% of the total variance (49.5%) in amino acid composition of chicken proteins. The distribution of the amino acid residues and the total genes for the first two axes is shown in figure 2. The first axis (Axis 1) accounts for 17.8% of the total variability, which is strongly correlated with the GRAVY score (general average hydropathicity) of proteins (r  =  0.7341, P <0.0001), Aromo score of proteins (r  =  0.5519, P <0.0001)(see figure 3), weakly correlated with the GC_cds_ (r  =  0.2653, P <0.0001), GC1(r  =  −0.0739, P  =  0.0151), GC2 (r  =  0.4609, P <0.0001), GC3(r  =  0.1912, P <0.0001), and negatively correlated with the gene expression level (r  =  −0.1471, P <0.0001). As shown in figure 2a, the strong hydrophobic amino acids, Ile, Val, Phe, and Met, except for Leu, and the aromatic amino acids, Tyr, Phe, and Trp, are at the right of the plane (positive values for axis 1). The distribution of genes in figure 2b indicated that the membrane proteins were related to the distribution of axis 1, in which the majority of them show a positive value over the axis 1.

**Figure 4:**
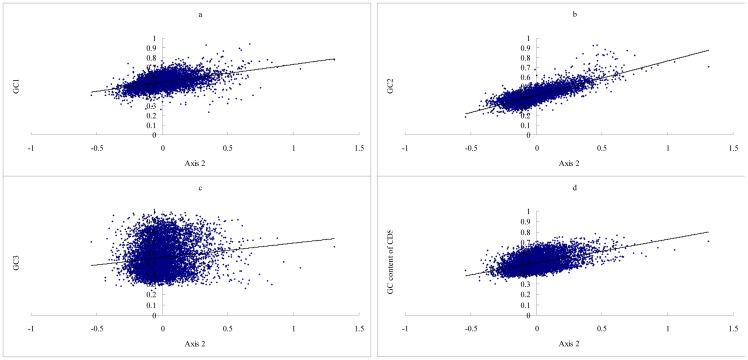
Relationship between Axis 2 and GC content. a. Axis 2 is positively correlated with GC1 significantly (r  =  0.4509, P <0.0001); b. Axis 2 is strongly correlated with GC2 positively (r  =  0.7782, P <0.0001); c. Axis 2 is weakly correlated with GC3 positively (r  =  0.1361, P <0.001); d. Axis 2 is positively correlated with the GC content of CDS (r  =  0.4608, P <0.0001).

**Figure 5:**
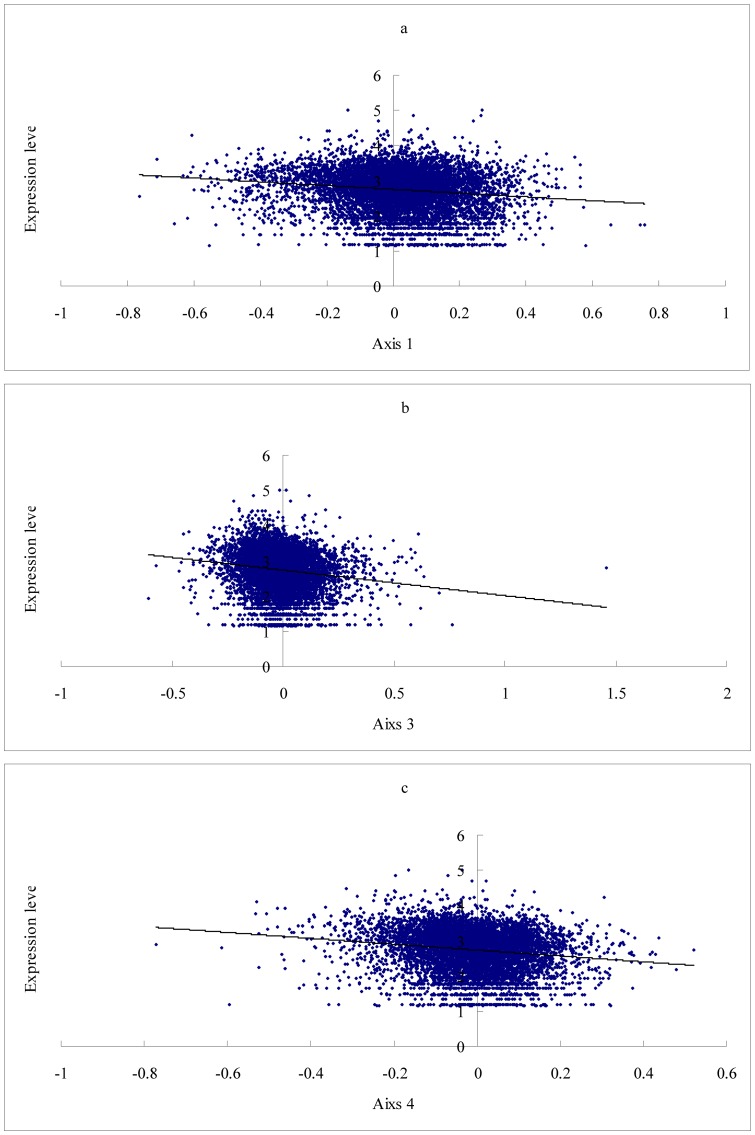
Relationship between gene expression level and Axis 1, Axis 3, and Axis4. Chicken expression data was taken from a previous work [20], including 19 tissues i.e. blood, brain, bursa of fabricius, cecum, connective tissue, embryonic tissue, epiphyseal growth plate, gonad, head, heart, limb, liver, muscle, ovary, pancreas, spleen, testis, and thymus. For a given gene, expression level is the number of EST counts in all tissues (transformed to denary logarithm). a. Axis 1 is negatively correlated with gene expression level (r  =  −0.1471, P <0.0001). b. Axis 3 is negatively correlated with gene expression level (r  =  −0.1664, P <0.0001); c. Axis 4 is negatively correlated with gene expression level (r  =  −0.1578, P <0.0001).

Axis 2 accounts for 15.2% of the total variability, which is positively correlated with the GC1(r  =  0.4509, P <0.0001), GC2 (r  =  0.7782, P <0.0001), GC3(r  =  0.1361, P <0.0001) and GC_cds_ (r  =  0.4608, P <0.0001), but with higher coefficients than Axis 1 except for GC3 (see figure 4). This axis also shows a negative correlation with the GRAVY score of proteins (r  =  −0.4550, P <0.0001) and the Aromo score of proteins (r  =  −0.5428, P<0.0001). The third and fourth axes represented 8.6%, and 7.9% of the total variability, respectively. Both axis 3 and axis 4 show a negative correlation with the gene expression level almost as well as does axis 1 (see figure 5. Axis 3 vs. expression level, r  =  −0.1664, P <0.0001; Axis 4 vs. expression level, r  =  −0.1578, P <0.0001). This implies that gene expression level also has somewhat impact on amino acid usage in chicken. Regression analyses among Axis 3, Axis 4 and the RAAU for each amino acid indicated that Axis 3 significantly correlated with the RAAU of Cys (r  =  0.6912, P <0.0001) and Ala (r  =  0.5391, P <0.0001), Axis 4 significantly correlated with the RAAU of Leu (r  =  0.5373, P <0.0001), Ser (r  =  0.4423, P <0.0001), and Gly (r  =  −0.4430, P <0.0001), suggesting that these amino acid usage are main contributors to Axis 3 and Axis 4 (see Figure S1 and Figure S2).

## Discussion

In the present study, we made a systematic study of the amino acid usage in the chicken proteome. Our data indicated that the relative amino acid usage is positively correlated with the tRNA gene copy number strongly. The isochore structure has significant impact on the amino acid usage. Correspondence analyses further indicated that the main factors responsible for the variation of amino acid usage are hydrophobicity, aromaticity, and genomic GC content.

Among microbes, as well as multicellular eukaryotes, a strongly positive correlation between amino acid usage and tRNA concentration, especially for highly expressed genes, has been documented, suggesting adaptation of both tRNA abundances and amino acid usage to enhancing the speed and accuracy of protein synthesis [2]–[8]. In yeast, Akashi [27] demonstrated that the correlation between tRNA concentrations and amino acid usage among highly expressed proteins is little stronger than that among less abundant proteins. The same trend also found in chicken in the present study.

Many studies have explored the relationship of the amino acid usage and genomic DNA properties, particularly in regard to genomic GC content, and have verified that the usage of amino acid types encoded by codons rich or poor in GC content are correlated with the genomic GC content significantly [1], [ 9]–[15]. A biologically relevant conclusion from the effect of GC content on amino acid usages is that differences in mutational biases could explain some of the variation in amino acid usage. Knight et al. [12] made a comparative study on the impact of GC content on codon usage and amino acid usage for bacteria, archaea and eukaryotes with limited gene sample. They concluded that codon responses are determined by the difference between its GC content and the mean GC content of its synomys (explaining 71–87% of the variance). Amino acid responses are determined by the mean GC content of their codons (explaining 71–79% of the variance). The prediction of above model is borne out qualitatively with the present study, but displays distinguished quantitative discrepancies. Indeed, our data demonstrated that GC content, including GC1, GC2, GC3,GC_cds_ and GC _introns_, was significantly correlated with the most of the amino acid usage, especially for GC rich and GC poor amino acids, however, multiple linear regression analyses indicated that only approximately 10–40% variation of amino acid usage can be explained by GC content for GC rich and GC poor amino acids. For other intermediate GC content amino acids, only approximately 10% variation can be explained. Our previous study in chicken codon bias indicated that the variation in the GC_cds_ could account for over 60% of the variation of codon usage, suggesting that mutation bias is the driving force of the codon usage in chicken [21]. The great difference between codon usage and amino acid usage influenced by GC content is more likely owing to the degeneracy of the genetic code; as a mutation occurred on the third position of codons do not necessarily lead to amino acid changes. In other words, the degeneracy of genetic code serves to significantly minimize the effects of mutation on amino acid usage.

Correspondence analysis of the amino acid usage indicated that hydrophobicity and aromaticity are the first factors shaping variation in amino acid usage among chicken proteins. As seen in Figure 2b, the majority of membrane proteins show a positive value over the axis 1. As the membrane proteins analyzed in this study only represent a small subset of the whole set of proteins. We would like to know whether the different amount of secondary structure among proteins also contributed to this variation. By the use of PHD software, we predicted the amount of secondary structure for each protein. Similar to previous studies [12], [28], our data demonstrated that Axis 1 correlated with the amount of alpha helix significantly (r  =  −0.4440, P <0.0001; see Figure S3). This finding could be expected, since hydrophobic residues tend to be more frequent in this secondary structure, especially for membrane proteins. We also found that Axis 1 weakly correlated with the amount of extended strand (r  =  0.1465, P <0.0001), and the amount of random coil (r  =  0.0765, P <0.0001). This means that protein secondary structures have significant impact on the amino acid usage in chicken. The second factor shaping variation in amino acid usage in chicken proteins is GC content, especially for GC1 and GC2. Amino acids are not evenly used in the chicken proteins. Some amino acids are overrepresented in the highly expressed genes, and others are overrepresented in the lowly expressed genes. Akashi and Gojobori [6] made a detailed study of metabolic efficiency in bacteria by analyzing the cost of each amino acid in terms of high-energy phosphate bonds, and found that the abundance of costly amino acids in highly expressed genes is significantly decreased, suggesting that a selection against highly energetically costal amino acids in highly expressed genes. Although chicken's amino acids are mainly obtained from the diet, some amino acids (essential amino acids) cannot be synthesized natively by themselves, the same trend indeed existed in chicken. For example, the aromatic amino acids, Trp, Phe, and Tyr, are the most expensive, and significant underrepresentation in highly expressed genes in our data set. We used 5% of the total genes with extremely high and low expression levels as the high and low expression data set and compared the average molecular weight of protein between two datasets using the one-way analysis of variance (ANOVA) by SAS. Our data indicated that the molecular weight of protein for highly expressed gene set is significantly lower than that of lowly expressed gene set (Fw _highly expressed_  =  40975.93 ± 3707.63, Fw _lowly expressed_  =  49031.52 ± 4707.88; F = 23.57, P  = 0.0116). In conclusion, our study demonstrated that the amino acid usage is related to the selection to maintain hydrophobic amino acids in integral membrane proteins, and against energetically costal amino acids such as aromatic amino acids in the highly expressed genes. A significant correlation between the relative amino acid usage and the tRNA abundance also suggested that translational selection contributes to enhancing the speed and accuracy of protein synthesis in chicken. Mutation also plays an important role in the amino acid usage; however, the effect of mutation on the amino acid usage is significantly lower than that of mutation on the codon usage. We argued that the amino acid usage of chicken proteome likely reflects a balance or near balance among selection, mutation, and genetic drift.

## Supporting Information

Figure S1 **Relationship between Axis 3 and the RAAU for Cys and Ala.** a. Axis 3 significantly correlated with the RAAU of Cys (r  =  0.6912, P <0.0001); b. Axis 3 positively correlated with the RAAU of Ala (r  = 0.5391, P <0.0001).
(TIF)

Figure S2 **Relationship between Axis 4 and the RAAU for Leu, Ser and Gly.** a. Axis 4 significantly correlated with the RAAU of Leu (r  =  0.5373, P <0.0001); b. Axis 4 significantly correlated with the RAAU of Ser (r  =  0.4423, P <0.0001); c. Axis 4 significantly correlated with the RAAU of Gly (r  =  −0.4430, P <0.0001).
(TIF)

Figure S3 **Relationship between Axis 1 and the amount of alpha helix.** Axis 1 significantly correlated with the amount of alpha helix (r  =  −0.4440, P <0.0001).
(TIF)
