# Supplementary figures and images for: Hydrophobicity and Aromaticity Are Primary Factors Shaping Variation in Amino Acid Usage of Chicken Proteome
Source: PLoS One. 2014 Oct 16;9(10):e110381. doi: 10.1371/journal.pone.0110381 (PMC4199684; doi:10.1371/journal.pone.0110381)

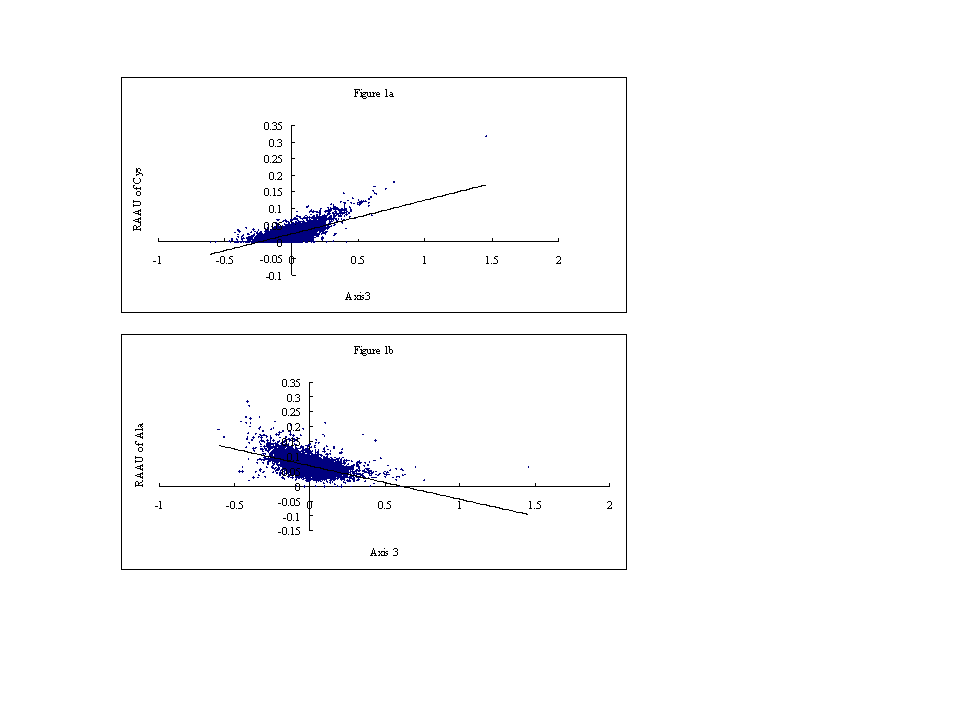

Supplement: Figure S1 — Relationship between Axis 3 and the RAAU for Cys and Ala. a. Axis 3 significantly correlated with the RAAU of Cys (r = 0.6912, P <0.0001); b. Axis 3 positively correlated with the RAAU of Ala (r = 0.5391, P <0.0001). (TIF) [file pone.0110381.s001.tif]

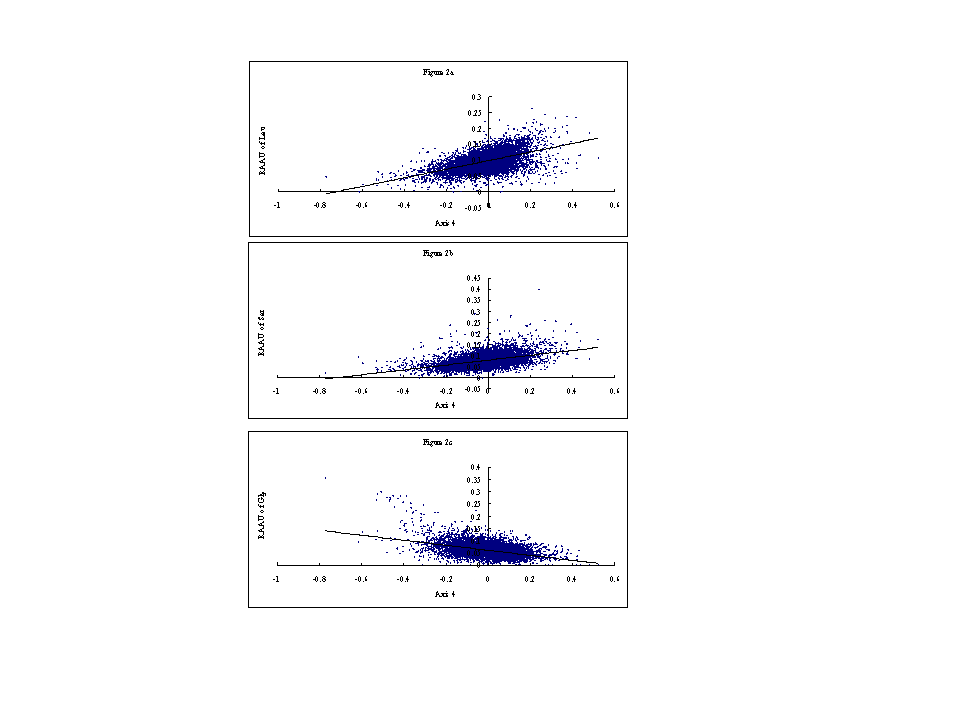

Supplement: Figure S2 — Relationship between Axis 4 and the RAAU for Leu, Ser and Gly. a. Axis 4 significantly correlated with the RAAU of Leu (r = 0.5373, P <0.0001); b. Axis 4 significantly correlated with the RAAU of Ser (r = 0.4423, P <0.0001); c. Axis 4 significantly correlated with the RAAU of Gly (r = −0.4430, P <0.0001). (TIF) [file pone.0110381.s002.tif]

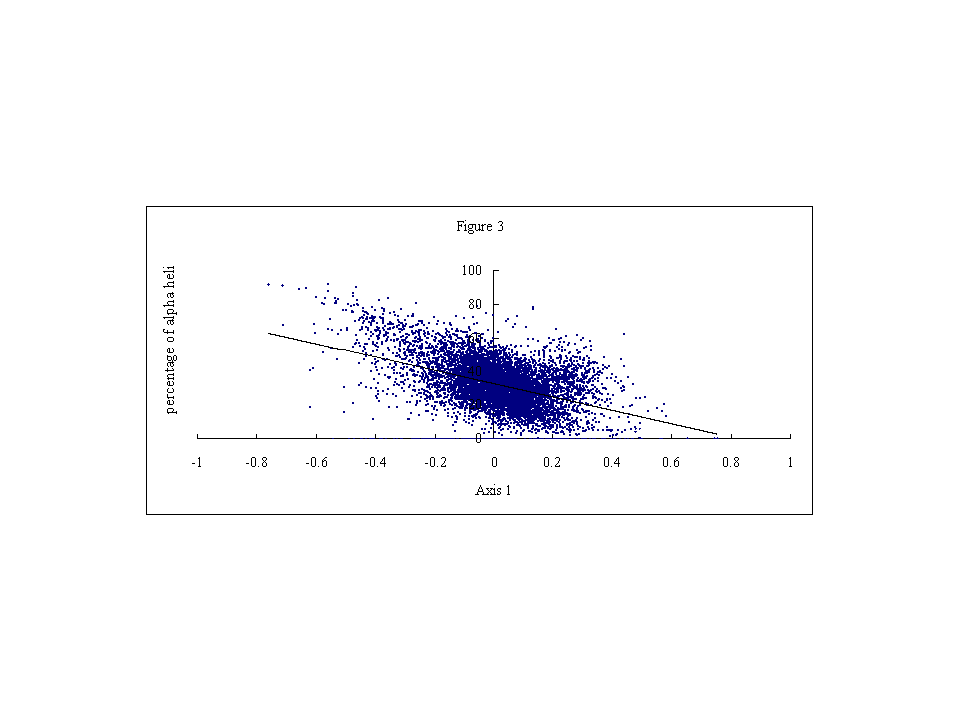

Supplement: Figure S3 — Relationship between Axis 1 and the amount of alpha helix. Axis 1 significantly correlated with the amount of alpha helix (r = −0.4440, P <0.0001). (TIF) [file pone.0110381.s003.tif]
